# Experimental dopaminergic neuron lesion at the area of the biological clock pacemaker, suprachiasmatic nuclei (SCN) induces metabolic syndrome in rats

**DOI:** 10.1186/s13098-021-00630-x

**Published:** 2021-01-23

**Authors:** Shuqin Luo, Michael Ezrokhi, Nicholas Cominos, Tsung-Huang Tsai, Carl R. Stoelzel, Yelena Trubitsyna, Anthony H. Cincotta

**Affiliations:** VeroScience LLC, 1334 Main Road, Tiverton, RI 02878 USA

**Keywords:** Dopamine, Suprachiasmatic nuclei, Biological clock, Hypertension, Sympathetic tone, Insulin resistance, Obesity, Metabolic syndrome

## Abstract

**Background:**

The daily peak in dopaminergic neuronal activity at the area of the biological clock (hypothalamic suprachiasmatic nuclei [SCN]) is diminished in obese/insulin resistant vs lean/insulin sensitive animals. The impact of targeted lesioning of dopamine (DA) neurons specifically at the area surrounding (and that communicate with) the SCN (but not within the SCN itself) upon glucose metabolism, adipose and liver lipid gene expression, and cardiovascular biology in normal laboratory animals has not been investigated and was the focus of this study.

**Methods:**

Female Sprague–Dawley rats received either DA neuron neurotoxic lesion by bilateral intra-cannula injection of 6-hydroxydopamine (2–4 μg/side) or vehicle treatment at the area surrounding the SCN at 20 min post protriptyline ip injection (20 mg/kg) to protect against damage to noradrenergic and serotonergic neurons.

**Results:**

At 16 weeks post-lesion relative to vehicle treatment, peri-SCN area DA neuron lesioning increased weight gain (34.8%, P < 0.005), parametrial and retroperitoneal fat weight (45% and 90% respectively, P < 0.05), fasting plasma insulin, leptin and norepinephrine levels (180%, 71%, and 40% respectively, P < 0.05), glucose tolerance test area under the curve (AUC) insulin (112.5%, P < 0.05), and insulin resistance (44%—Matsuda Index, P < 0.05) without altering food consumption during the test period. Such lesion also induced the expression of several lipid synthesis genes in adipose and liver and the adipose lipolytic gene, hormone sensitive lipase in adipose (P < 0.05 for all). Liver monocyte chemoattractant protein 1 (a proinflammatory protein associated with metabolic syndrome) gene expression was also significantly elevated in peri-SCN area dopaminergic lesioned rats. Peri-SCN area dopaminergic neuron lesioned rats were also hypertensive (systolic BP rose from 157 ± 5 to 175 ± 5 mmHg, P < 0.01; diastolic BP rose from 109 ± 4 to 120 ± 3 mmHg, P < 0.05 and heart rate increase from 368 ± 12 to 406 ± 12 BPM, P < 0.05) and had elevated plasma norepinephrine levels (40% increased, P < 0.05) relative to controls.

**Conclusions:**

These findings indicate that reduced dopaminergic neuronal activity in neurons at the area of and communicating with the SCN contributes significantly to increased sympathetic tone and the development of metabolic syndrome, without effect on feeding.

## Introduction

Many vertebrate species in the wild exhibit annual cycles of metabolism, oscillating between seasons of obese, insulin resistance and lean, insulin sensitivity [1, 2]. The ability to anticipate a season of low food availability by the endogenous induction of the obese, insulin resistant state supports survival during such a subsequent season when food availability is scarce. Physiological studies of seasonal animals have established important roles for interactions of circadian rhythms of neuroendocrine events in the manifestation of seasonal physiology, including metabolism. The entire seasonal repertoire of metabolic events in representative species among teleost, avian, and mammalian vertebrate classes can be induced in animals maintained on 24-h constant light conditions by varying the circadian-time of administration of levo-3,4, dihydroxyphenylalanine (L-DOPA), the precursor to dopamine, relative to the circadian-time of administration of 5-hydroxytryptophan (5HTP), the precursor to serotonin over an approximate ten day treatment period [3–5]. That is, the response to L-DOPA functions at one circadian time of day relative to a static timed administration of 5HTP, to induce seasonal obesity while it functions at another circadian time of day relative to the same static timed administration of 5HTP to induce the seasonal lean condition.

Inasmuch as the suprachiasmatic nuclei (SCN) are the seat of the circadian pacemaker system of the vertebrate body that function via the neuroendocrine axis to synchronize temporal biology (e.g., daily metabolism) with the cyclic environment, it was postulated that such L-DOPA effects were acting at least in part by modulating SCN output function [4]. It was subsequently observed that the circadian peak input of dopamine release at the SCN differs in seasonal obese, insulin resistant and seasonal lean, insulin sensitive rodents. The circadian peak of dopamine release at the peri-SCN area in seasonal lean, insulin sensitive animals (at the onset of locomotor activity) was markedly diminished in naturally occurring (seasonally) obese, insulin resistant animals and also in obesogenic diet-induced glucose intolerant animals [6]. However, whether such reduction in peri-SCN dopaminergic input signaling is causal in the induction and maintenance of the insulin resistance syndrome, a constellation of pathologies of insulin resistance, obesity, and hypertension has never been evaluated. Hypertension is a common correlate of obesity [7] and elevated sympathetic tone is a common pathophysiological condition associated with both hypertension and the hyperinsulinemic/insulin resistant obese condition in animals and humans [8]. More importantly, such increased sympathetic nervous system (SNS) activity is a potent stimulus for development of insulin resistance syndrome, type 2 diabetes, and cardiovascular disease [9–13], currently the most prevalent diseases on earth [14]. The cause-effect relationship between elevated SNS tone and insulin resistance syndrome remains poorly understood inasmuch as a positive feedback loop exists between these two pathophysiologies [15]. However, a common CNS neurologic contributory factor/circuitry for both the insulin resistance syndrome and elevated SNS tone may include the SCN. The SCN is a major control center for regulation of the autonomic nervous system (ANS) as it communicates with and regulates the output of preautonomic neuronal centers in the brain (e.g., the paraventricular nucleus [PVN], the ventromedial nucleus [VMH], the arcuate nucleus [ARC]), projections from which impinge on and regulate preganglionic neurons of the ANS that regulate both metabolism and vascular SNS tone [16–23]**.** However, what factors regulate SCN output signals to modulate both metabolism and vascular tone, remains incompletely defined. Given the association of diminished circadian peak dopaminergic activity at the peri-SCN area with insulin resistance and the presence of dopamine D1 and D2 receptors in the SCN and peri-SCN regions [24], we hypothesized that a chronic diminution of dopaminergic input activity to the peri-SCN/SCN clock area is actually operative in facilitating the obese, hyperinsulinemic/insulin resistant and hypertensive state. We therefore investigated this major question by assessing the metabolic (glucose tolerance, insulin sensitivity, plasma leptin level, adipose and liver lipid metabolism genes expression, body fat level) and vascular hemodynamic (blood pressure, heart rate, and circulating norepinephrine level) impact of dopaminergic neuron lesion at the peri-SCN area (via neurotoxic lesion) in young, female Sprague–Dawley rats, a model that maintains normal body fat, glucose metabolism and vascular biology for a majority of its lifespan.

## Materials and methods

### Animals

Female Sprague–Dawley rats obtained from Taconic Biosciences (Hudson, NY) (where they are routinely bred and maintained on 12-h daily photoperiods for extended generations [Taconic Biosciences]) were used in these studies. Such animals at 10 weeks of age; (body weight 220 ± 3 g) were maintained on long 14-h daily photoperiods (14hours light/10 h dark) typical of the summer lean, insulin sensitive season in temperate zone rodents [1] and allowed to feed regular rodent chow (2018 Teklad rodent diet, Envigo, East Millstone, NJ) ad libitum. To avoid the confounder of age-induced insulin resistance upon the background metabolic status of the study animals during the long duration of the study time period, female rats were used since they maintain a steady state of insulin sensitivity from an early age for a long period of their lifetime versus male rats of this strain that develop insulin resistance progressively from an early age [25, 26]. Potential influence of estrous cycle day on study parameter outcomes was minimized by random daily investigation of study endpoints across random animals within the study groups over a 7-day time period. However, it has previously been observed that the estrous cycle day does not influence hypothalamic glucose sensing response to elevated (post-meal) glucose levels to impact insulin mediated glucose disposal [27]. Rats were habituated to our climate-controlled animal facilities for at least 7 days before initiation of any experimentation.

### Experimental design

Two separate studies were conducted to assess the impact of peri-SCN area dopamine neuron lesion on peripheral glucose tolerance, insulin sensitivity, adipose and liver lipid metabolism gene profile, obesity, and vascular biology. In Study 1, rats were randomly assigned to one of two treatment groups (N = 8/group) and infused bilaterally at the peri-SCN area with either vehicle or the dopamine neurotoxin, 6-hydroxydopamine (6-OHDA) at 8 μg/side 20 min following systemic intraperitoneal administration of protriptyline (20 mg/kg, i.p.) to protect norepinephrine and serotonin neurons. Then, an intraperitoneal glucose tolerance test (GTT) was performed at 16 weeks following the lesion to examine any effect on peripheral glucose metabolism and insulin sensitivity during the GTT. Body weight change from baseline was also obtained. In Study 2, based upon the results of Study 1, animals were similarly treated as in Study 1 with the exceptions that the 6-OHDA dose was lowered to 2–4 μg/side (there was no significant metabolic response difference between 2 and 4 μg/side 6-OHDA doses, and data were combined for analysis vs vehicle control), GTT data were obtained at both 8 and 16 weeks following lesion, and measures of humoral factors regulating metabolism, adipose and liver metabolic gene expressions, body fat store levels, and vascular biology (blood pressure and heart rate) were also taken at week 16. The treatment group (N = 14) received an infusion of dopaminergic neurotoxin into the peri-SCN area bilaterally; the other group (N = 8) received a vehicle infusion. Intraperitoneal GTTs were performed 8 and 16 weeks after the neurotoxin infusion. Blood pressure and heart rate were measured after two days recovery from the GTT at 16 weeks. Body weight and food consumption were monitored during the course of the study. Animals were sacrificed after the vascular biology assessments and blood samples were collected for analyses of humoral metabolic factors, including plasma insulin, glucose, norepinephrine (NE) and leptin. Parametrial and retroperitoneal fat pads were removed and weighed as an index of body fat store level. Adipose and liver tissues were stored at -80 ^0^C for analyses of lipid metabolism gene expressions.

A separate histological study was conducted to verify the viability of the SCN neurons several days following such peri-SCN 6-OHDA treatment. Also, a separate study was conducted to verify the specific peri-SCN dopaminergic neuronal lesion several days following such peri-SCN administration of 6-OHDA. All animal experiments were conducted in accordance with the National Institutes of Health Guide for the Care and Use of Experimental Animals (2011) and also with the protocols approved by the Institutional Animal Care and Use Committee of VeroScience, LLC.

### Peri-SCN 6-OHDA infusion impact on SCN neurons

Four weeks after peri-SCN neuron lesioned (4 or 8 µg/side 6-OHDA plus protriptyline 20 mg/kg i.p. to protect norepinephrine and serotonin neurons) rats were sacrificed by decapitation. Their brains were quickly collected, and postfixed in buffered formalin and then transferred to buffered sucrose solution, frozen, and sliced into 50-mm coronal sections through SCN in a cryostat. Sections were mounted on a gelatin-coated slide and stained with cresyl violet to assist in evaluation of the lesions post infusion of 6-OHDA.

### Peri-SCN 6-OHDA infusion selective impact on peri-SCN dopaminergic neurons

Animals were randomly divided into three groups (N = 8/group). Following 6-OHDA neurotoxin injection to the peri-SCN area (at a dose of either 2 or 8 µg/SCN side) 20 min following systemic intraperitoneal administration of protriptyline [20 mg/kg, i.p.] or vehicle treatment to the peri-SCN area as described below, a 30-gauge stainless steel microdialysis guide cannula was stereotaxically implanted at the right side of SCN with coordinates: 1.3 mm posterior to bregma, 0.4 mm right side of lateral to the midsagittal suture, and 8.4 mm ventral to the dura. The cannula was anchored firmly to the skull with stainless steel screws and secured to the surface with dental cement. Microdialysis experimentation was conducted after 12 to 20 days of lesioning. During the test days, each animal was placed in an acrylic bowl with free access to food and water. A 32-gauge dialysis probe with a 1-mm-long tip of semi-permeable membrane (20,000 molecular weight cutoff) was inserted into the guide cannula and the probe membrane protruded 1 mm outside the guide cannula. Using a microinjection pump (CMA/100), Cerebral Spinal Fluid (CSF) solution was continuously perfused through the probe at a rate of 0.5 µl/min. The probe was connected to the microinjection pump by micrbore Teflon tubing through a counterbalanced 2-channel liquid swivel arm (Bioanalytical Systems, West Lafayette, IN, USA) attached to the rim of the bowl, thus permitting the animal to move freely without the tubing becoming tangled during the experimental period. Dialysate collection began 2 h after insertion of the probe to allow some recovery from potential tissue damage by probe insertion. Samples were collected into 300 µl glass vials (containing 2 µl of 0.1 N perchloric acid solution) at 30 min intervals through an automated refrigerated fraction collector (modified CMA/170, CMA microdialysis, MA) over a 3 h period while animals were maintained on a 14-h daily photoperiod and allowed free access to food and water. The 10 µl dialysis samples were analyzed immediately by HPLC with electrochemical detection (Coulochem III electrochemical detector, ESA, Chelmsford, MA), as described previously [24]. Dopamine (DA) and DA metabolites (3,4-dihydroxyphenylacetic acid [DOPAC], homovanillic acid [HVA]), NE and NE metabolite (3-methoxy-4-hydroxyphenylglycol [MHPG]), and the serotonin metabolite (5-hydroxyindoleacetic Acid [5-HIAA]) in microdialysis samples were measured. All probes were tested for recovery in vitro prior to use and data were adjusted for recovery rate.

### Peri-SCN dopamine neuron-selective lesion with 6-OHDA

**Fig. 1:**
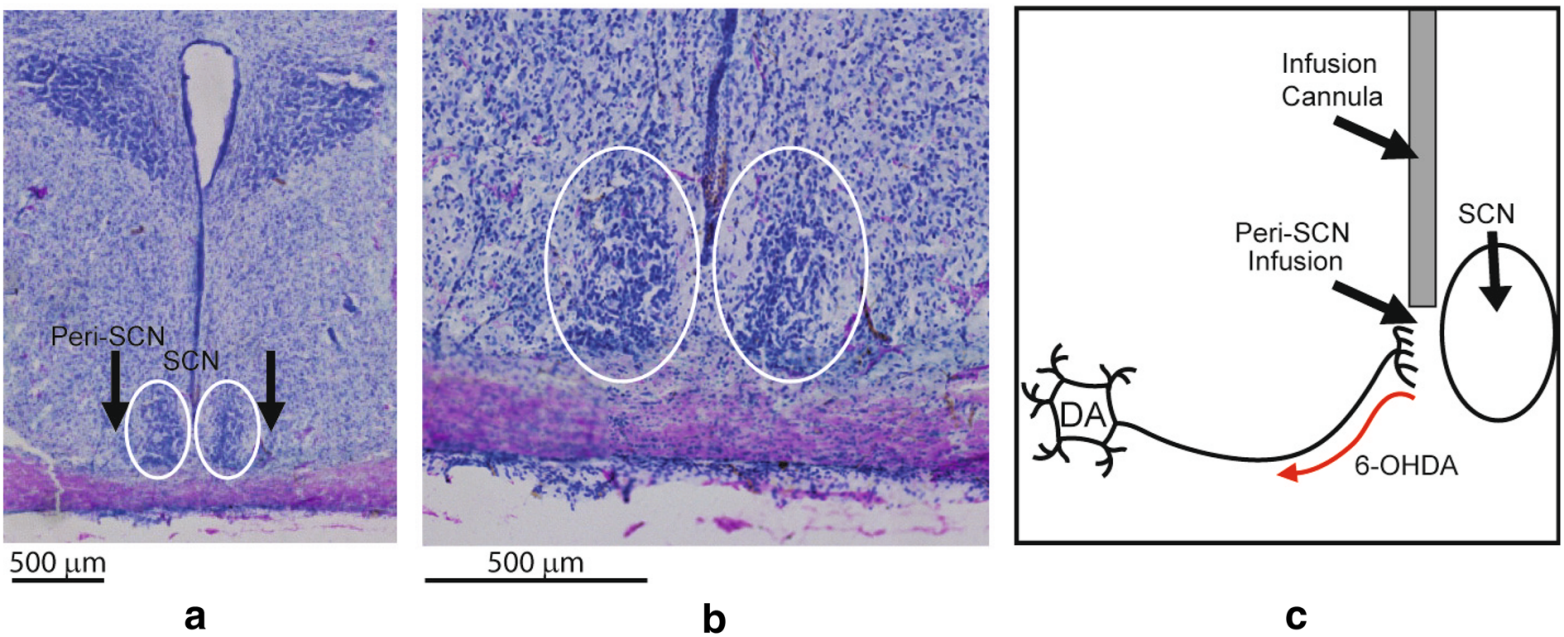
Histology of SCN at 8 weeks post infusion of 6-OHDA**.** The external cannula tip location was at the stereotaxic coordinates of anteroposterior−1.3, mediolateral 0.4 mm and dorsoventral−7.4 mm from dura. The injection cannula protruded 2 mm from the guide cannula landing lateral to the perimeter edge of the SCN. A representative picture of a peri-SCN coronal section from a dopamine neuron lesioned (4 µg/side 6-OHDA plus protriptyline 20 mg/kg ip) rat is shown stained with cresyl violet with placement of the infusion cannula (**a**). The arrows indicate the infusion sites just outside the SCN (peri-SCN area). **b** An enlarged micrograph of the same histology section of (**a**) of the peri-SCN and SCN regions. The cresyl violet staining demonstrates normal neuronal anatomy with no neuronal damage. **c** Diagrammatic representation of the peri-SCN injection target site. The 6-OHDA is taken up by dopamine neuron terminals in the peri-SCN region and transported back to the cell body where it is toxic to the cell

6-OHDA neurotoxin treatment to the peri-SCN area was performed in a manner to damage only dopaminergic neuronal input to the SCN without damaging neurons within the SCN itself (see Results, Fig. 1). Each animal was anesthetized with ketamine/xylazine (60/5 mg kg^−1^ body weight, i.p.) and placed in a stereotaxic frame (David Kopf). A double stainless steel guide cannula was implanted at coordinates 1.3 mm anterior to bregma, 0.4 mm each side of lateral to the midsagittal suture, and 7.4 mm ventral to the surface of the skull (landing at 2 mm above SCN). The injection cannula (33-gauge) inserted through the guide cannula extended to a total depth of 9.4 mm. 6-OHDA was infused bilaterally to the peri-SCN area of each animal to selectively damage or destroy dopaminergic neuron terminals outside of the SCN, twenty minutes after each animal received an intraperitoneal injection of protriptyline (20 mg/kg, i.p.) to block neurotoxic effects of 6-OHDA to noradrenergic and serotonergic neurons [28]. This is a well established method to selectively damage dopaminergic neurons without affecting noradrenergic or serotonergic neurons [28]. Although there are dopamine D2 and D1 receptors and amino acid decarboxylase within the SCN itself, there are no tyrosine hydroxylase positive neuronal cell bodies within the nucleus [24], thus precluding damage to SCN neruons with this procedure. In Study 1, rats were subjected to infusion of either 6-OHDA (8 μg/side, N = 8) in saline containing 0.2% ascorbic acid or vehicle (saline containing 0.2% ascorbic acid, N = 8) at the peri-SCN area bilaterally. The intra-cannula infusion was carried out over 2 min at a flow rate of 0.2 µl/min (a total injection volume of 0.4 µl for each side of SCN). A further 60 s was allowed after the infusion for the solution from the tip of the cannula to diffuse into the peri-SCN area. In Study 2, rats were similarly handled, prepared and treated with 6-OHDA infusion at the peri-SCN area at 2–4 μg/side (N = 14) or vehicle (N = 8).

### Glucose tolerance test

Glucose tolerance tests were performed 5 h after light onset at 16 weeks following the SCN 6-OHDA lesion in Study 1 or at 8 and 16 weeks following the peri-SCN area 6-OHDA lesion in Study 2. A 50% glucose solution was administered intraperitoneally (3 g/kg body weight) and blood samples were taken from the tail before glucose administration and 30, 60, 90, 120 min after glucose injection. Blood samples were collected into vials with 5 µl EDTA and were immediately separated by centrifugation and stored at -80 C until assay of insulin. Matsuda index [29] was calculated to assess insulin sensitivity. The Matsuda index was calculated as follows: Matsuda index = 10,000/sqrt {0 min (before loading) plasma glucose (mg/dL) × 0 min serum insulin (μU/mL) × 120 min plasma glucose × 120 min serum insulin}.

### Assay of metabolic parameters

Blood glucose concentrations were determined at the time of blood collection by a blood glucose monitor (OneTouch Ultra, LifeScan, Inc; Milpitas, CA, USA). Plasma insulin, leptin and NE were assayed by an enzyme immunoassay using commercially available assay kits utilizing anti-rat serum and rat insulin, leptin and NE as standards (ALPCO Diagnostics, Salem, NH, USA).

### Assay of adipose lipid metabolism genes expression

Total RNA was isolated from frozen parametrial adipose tissue samples utilizing Trizol Reagent (ThermoFisher). Total RNA quantity and purity was determined by UV spectroscopy and the concentration was normalized prior to reverse transcription reaction. Reverse transcription was performed with iScript RT Supermix for RT-qPCR (BioRad), followed by qPCR. The mRNA quantities of studied genes were assessed with use of the following probes: hormone sensitive lipase (HSL) was determined with ThermoFisher Assay Rn00689222, phosphoenolpyruvate carboxykinase (PEPCK1) was determined with ThermoFisher Assay Rn01529014, phosphoenolpyruvate carboxykinase 2, (mitochondrial PEPCK2) was determined with ThermoFisher Assay Rn03648110, fatty acid synthase (FAS) was determined with ThermoFisher Assay Rn01463550, acetyl-CoA carboxylase 1 (ACC1) was determined with ThermoFisher Assay Rn01456588 and SsoAdvanced Universal Probes Supermix (BioRad). Pyruvate dehydrogenase complex x (PDHX), glucose 6-phosphate dehydrogenase (G6PDH), malic enzyme (ME1), and mitochondrial ATP synthase complex F1 (ATPF1) were quantified with PerfeCTa^®^ SYBR^®^ Green FastMix^®^ Low ROX QPCR Master Mix (Quantabio) on AriaMX qPCR instrument with the following primer pairs (Sigma Aldrich):

ATPF1 Forward: 5’ CTGGCGACAGGCTGGAC
ATPF1 Reverse: 5’ TGCTTCACCTGGAAGACTCC
G6PD Forward: CCTGATGCCTATGAACGCCT
G6PD Reverse: 5’CCCTCATACTGGAAGCCCAC
PDHX Forward: 5’TACTGTGCCTCACGCCTATG
PDHX Reverse: 5’GATGCTTTGGGCCTTCTCCA
ME1 Forward: 5’ ATGGAGAAGGAAGGTTTATCAAAG
ME1 Reverse: 5’ GGCTTCTAGGTTCTTCATTTCTTC

### Assay of liver lipid metabolism genes expression

Total RNA was isolated from frozen liver tissue samples with Trizol Reagent (ThermoFisher). Total RNA quantity and purity was determined by UV spectroscopy and concentration was normalized prior to reverse transcription reaction. Reverse transcription was performed with iScript RT Supermix for RT-qPCR (BioRad), followed by qPCR. The mRNA quantity of G6PDH, mitochondrial ATPF1, FAS, Carnitine O-Palmitoyltransferase 1 (CPT1), ACC1, Tumor necrosis factor α (TNFα), Monocyte Chemoattractant Protein 1 (MCP1) were quantified with PerfeCTa^®^ SYBR^®^ Green FastMix^®^ Low ROX QPCR Master Mix (Quantabio) on AriaMX qPCR instrument with the following primer pairs (Sigma Aldrich):

G6PDH Forward: 5′CCTGATGCCTATGAACGCCT
G6PDH Reverse: 5′CCCTCATACTGGAAGCCCAC
ATPF1 Forward: 5′CTGGCGACAGGCTGGAC.
ATPF1 Reverse: 5′TGCTTCACCTGGAAGACTCC
FASN Forward: 5′AATATATTGAAGCCCATGGCA
FASN Reverse: 5′GCCCAAACCCCATTTTCT
CPT1 Forward: 5′TCGTGGTGGTGGGTGTGAT
CPT1 Reverse: 5′AGCAGCACCTTCAGCGAGT
ACC1 Forward: 5′GATTCATAATTGGGTCCGTGTCT
ACC1 Reverse: 5′CTAGGTGCAAGCCGGACAT
TNFα Forward: 5′GTAGCCCACGTCGTAGCAAA
TNFα Reverse: 5′AAATGGCAAATCGGCTGACG
MCP1 Forward: 5′TAGCATCCACGTGCTGTCTC
MCP1 Reverse: 5′GAGCTTGGTGACAAATACTACAGC

### Blood pressure and resting heart rate measurements

Systolic and diastolic blood pressure (BP) and resting heart rate (RHR) were non-invasively measured by determining the tail blood volume with a volume pressure recording sensor and an occlusion tail-cuff (CODA-6 non-invasive blood pressure system, Kent Scientific Corp. Torrington, CT) in conscious rats during the animals’ normal sleeping period of the day (5 h after light onset) following the manufacturer’s instructions. Several days before experimental recordings, rats were acclimated to the restraining cage and the tail cuff to minimize or reduce any stress influence on the readings. BP and heart rate values per animal were the result of an average of 6–8 measurements.

### Statistical analysis

All date are expressed as mean ± SEM. Data comparing the group mean values of the plasma glucose and insulin during GTTs, and neurotransmitter levels in the microdialysis experitment were analyzed by two-way repeated measures ANOVA or one-way ANOVA as appropriate, followed by Duncan’s New Multiple range tests. Differences in the group mean values of the weight change from baseline, areas under the glucose or insulin curves during GTTs, fasting plasma insulin, glucose, NE or leptin levels, blood pressures, and hear rate were each determined by unpaired t-tests. A statistical value of P < 0.05 (2-tailed) was considered statistically significant.

## Results

### Effect of peri-SCN area 6-OHDA infusion on SCN viability

With the placement of the infusion cannula targeted at 0.4 mm lateral of the midline, just outside the SCN as in all 6-OHDA infusion studies conducted herein, neither the cannula nor the infusion of 6-OHDA caused damage to the SCN itself as observed from histological examination of the SCN and surrounding area 4 weeks following the treatment (see Fig. 1). A representative coronal section from a peri-SCN dopamine neuron lesioned (4 µg/side 6-OHDA plus protriptyline 20 mg/kg ip) rat brain is shown in Fig. 1. The cresyl violet staining demonstrates normal neuronal anatomy with no damage at the peri-SCN infusing area.

### Effect of peri-SCN 6-OHDA infusion on peri-SCN dopaminergic neuronal activity.

**Fig. 2:**
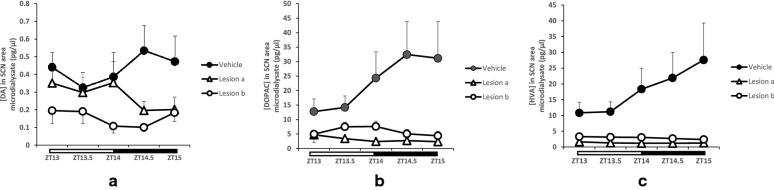
Daily profiles of extracellular dopamine, 3,4-dihydroxyphenylacetic acid, and homovanillic acid concentration (pg/μl) in microdialysate samples from the peri-SCN of freely-living rats (mean ± SEM, n = 8/group). Animals received either an infusion of 6-OHDA (2 or 8 μg/SCN side) or vehicle at the peri-SCN area bilaterally. Microdialysis experimentation was conducted at 12—20 days after lesion and samples were collected for each of 5 sequential 30 min intervals. The horizontal bar indicates light and dark phases of the time period samples were collected. **a** a two-way ANOVA with repeated measures on extracellular dopamine (DA) indicates an overall group effect (F_2, 21_ = 3.759, P < 0.05); Post hoc Duncan test shows a significant reduced extracellular DA in animals with each dose of 6-OHDA lesion compared to sham lesion. **b**, **c** Two-way ANOVA with repeated measures on extracellular DOPAC (**b**) and HVA (**c**) in peri-SCN reveals a significant group effect (F_2, 21_ = 5.291, F_2, 21_ = 5.378 respectively, P < 0.05). Both high and low 6-OHDA lesion markedly reduced extracellular DOPAC and HVA concentration at the peri-SCN. There was no difference in effect between low and high 6-OHDA dose lesion groups

At 12–20 days after the peri-SCN area 6-OHDA infusion at either 2 or 8 μg/side, peri-SCN extracellular dompaminergic activities were significantly reduced by 35% and 64% in animals with low and high dose of 6-OHDA lesion (Fig. 2). Two way ANOVA with repeated measures on DA indicates a significant group effect (F_2, 21_ = 3.759, P < 0.05). Post hoc Duncan test shows reduced extracellular DA in animals with low and high dose of 6-OHDA lesion compared to controls (P < 0.05, Fig. 2a). There was also a significant group effect of 6-OHDA upon both the extracellular DA metabolites DOPAC and HVA (Two-way ANOVA with repeated measures: F_2, 21_ = 5.378, P < 0.05, F_2, 21_ = 5.291, P < 0.05 respectively). Both 6-OHDA 2 and 8 ug/SCN side lesioned groups show greately reduced extracellular DA metabolite DOPAC (84 and 74%, respectively) and HVA (93 and 84%, respectively), compared to vehicle group (P < 0.05). There was no difference in treatment effect between the two different dosed lesioned groups. Such 6-OHDA infusion at either 2 or 8 ug/SCN side did not significantly affect peri-SCN NE (F_2, 21_ = 1.969, P = 0.166), it’s metabolite MHPG (F_2, 21_ = 0.814, P = 0.457), or serotonin metabolite 5-HIAA (F_2, 21_ = 1.981, P = 0.167) levels relative to controls.

### Study 1

**Fig. 3:**
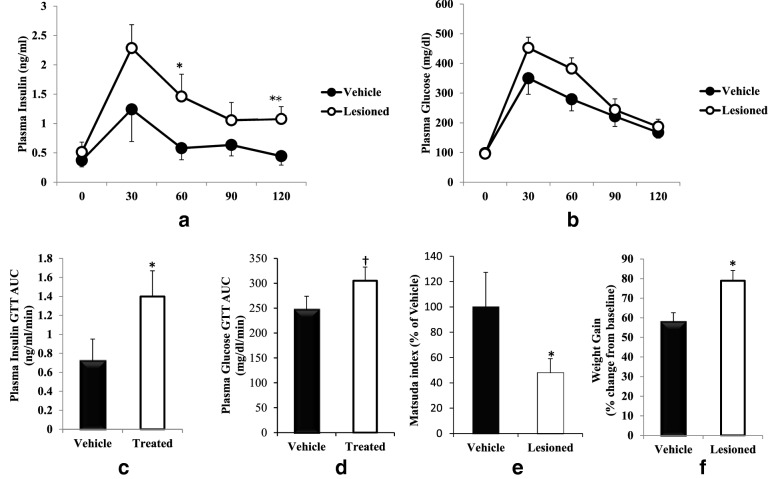
Peri-SCN area dopaminergic neuron lesion induced glucose intolerance. Animals received an infusion of 6-OHDA (8 μg) at the peri-SCN area bilaterally. A GTT was performed at 16 weeks after either 6-OHDA or sham lesion at 6 h after light onset. Plasma glucose and insulin levels were monitored before and at 30, 60, 90 and 120 min after glucose (3 g/kg BW, i.p.) was administered. The figure depicts the GTT plasma insulin (**a**), GTT plasma glucose (**b**) the area under the insulin (**c**) and glucose (**d**) GTT curve and Matsuda insulin sensitivity index derived from the GTT (**e**), and body weight gain at the end of the study (**f**). Data are expressed as mean ± SEM (n = 8 for each group). *P < 0.05 or ** P < 0.01 significant difference between treatment groups (two-way ANOVA with repeated measures followed by Student’s t-test, or unpaired Student’s t-test as appropriate)

Body weights of the two groups of rats at the start of experiment were 204.3 ± 6.1 (vehicle control) and 207.7 ± 2.1 (neurotoxin group). At 16 weeks following the peri-SCN area 6-OHDA infusion at a dose of 8 μg/side, the total area under the insulin GTT curve was increased by 95% (from 0.72 to 1.40 ng/ml/min, P < 0.05) without changing the GTT glucose AUC (p < 0.05 one tail), compared with vehicle peri-SCN area treated controls (Fig. 3a–d). Such 6-OHDA peri-SCN area treatment reduced insulin sensitivity by 52% (Matsuda Index, P < 0.05 one tailed) relative to control animals (Fig. 3e). Body weight gain over the study period in the peri-SCN area dopamine lesioned group was 37% greater than that among the sham controls (79% increase vs 58% increase, P < 0.02, Fig. 3f).

### Study 2

**Fig. 4:**
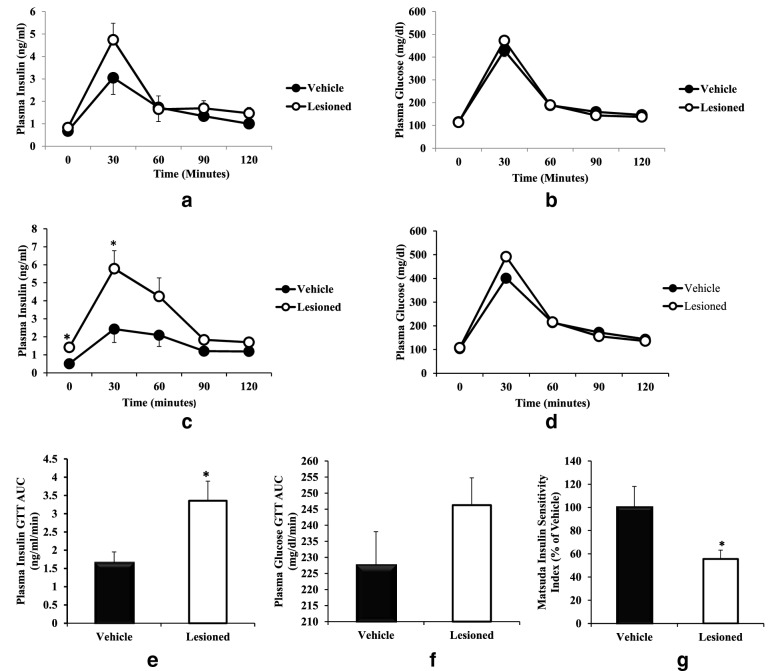
Peri-SCN area dopaminergic neuron lesion induced metabolic syndrome. Animals received an infusion of 6-OHDA (2–4 μg/side) at the peri-SCN area bilaterally. GTTs were performed at 6 h after light onset. Plasma glucose and insulin were monitored before and at 30, 60, 90 and 120 min after glucose (3 g/kg BW, i.p.) was administered. The figure depicts plasma insulin and glucose during a GTT performed at 8 weeks (**a**, **b** respectively) and a second GTT performed at 16 weeks (**c**, **d** respectively) after lesion (n = 14) or sham lesion (n = 8). The area under the insulin (**e**) and glucose (**f**) tolerance test curves and Matsuda insulin sensitivity index during the second GTT (**g**) at week 16. Data are expressed as mean ± SEM. * P < 0.05 or ** P < 0.01 significant difference between treatment groups (two-way ANOVA with repeated measures with post-hoc Student’s t-test, or unpaired Student’s t-test as appropriate).

**Fig. 5:**
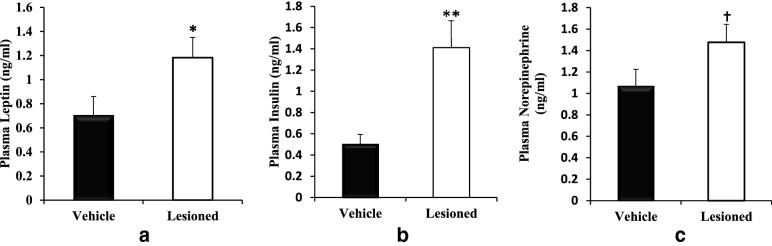
Peri-SCN area dopaminergic neuron lesion increased plasma leptin (**a**), plasma insulin (**b**) and plasma norepinephrine (**c**). Animals received an infusion of 6-OHDA (2–4 μg/side) at the peri-SCN area bilaterally, data were collected 17 weeks post-lesion. Data are represented as the mean ± SEM. * Significant difference between treatment groups (P < 0.05, unpaired Student’s t-test). †*P* < 0.05, 1-tailed t test

Body weights of the two groups of rats at the start of experiment were 219 ± 5 (vehicle control) and 221 ± 2 (neurotoxin group). At 8 weeks after the peri-SCN area 6-OHDA infusion at 2–4 μg/side, no treatment effect upon the GTT was demonstrable versus control (Fig. 4a–b). However, peri-SCN area DA neuron lesion significantly increased plasma insulin (P < 0.05, Fig. 4c), but not glucose (Fig. 4d) during the GTT at 16 weeks following the lesion, compared with vehicle control. The total stimulated area under the insulin GTT curve was increased by 113% (from 1.6 ± 0.3 to 3.4 ± 0.5 ng/ml/min, P < 0.05) (Fig. 4e), without change in GTT glucose AUCcompared with vehicle infusion during GTT (Fig. 4f). Insulin sensitivity was reduced by 44% (Matsuda Index, P < 0.02) in peri-SCN area dopamine neuron-lesioned animals relative to control animals (Fig. 4g). Peri-SCN area DA neurotoxin also increased fasting plasma insulin by 180% (from 0.5 ± 0.1 to 1.4 ± 0.3 ng/ml, P < 0.02, Fig. 5b), plasma leptin by 71% (from 0.7 ± 0.1 to 1.2 ± 0.2 ng/ml, P < 0.02, Fig. 5a), and plasma NE by 40% (from 1.1 ± 0.1 to 1.5 ± 0.2 ng/ml, P < 0.05, Fig. 5c) at 5 h after light onset in peri-SCN area DA lesioned animals compared to control animals.

**Fig. 6:**
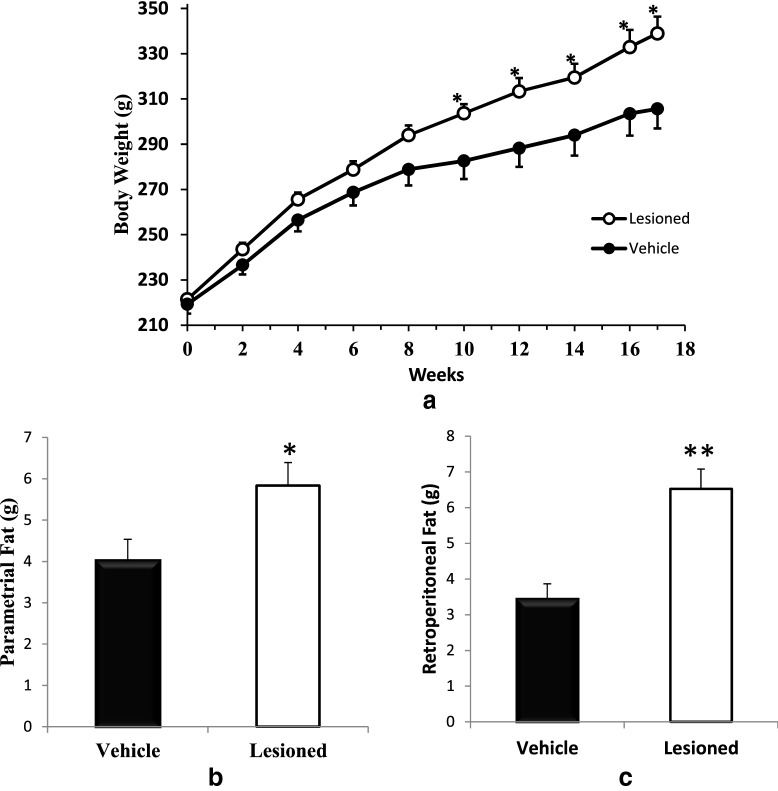
Peri-SCN area dopaminergic lesion increased body weight gain and abdominal body fat. Animals received an infusion of 6-OHDA (2–4 μg/side) at the peri-SCN area bilaterally. Body weight over 17 weeks after 6-OHDA lesion (n = 14) or vehicle sham lesion (n = 8) to SCN area (**a**). Parametrial fat pad weight (**b**) and retroperitoneal fat pad weight (**c**) at the end of experiment (week 17). Data are represented as the mean ± SEM. * P < 0.05 or ** P  < 0.01 significant difference between treatment groups (unpaired Student’s t-test)

Dopaminergic neurotoxin infusion to the peri-SCN area resulted in an increase in weight gain of 34.8% at 16 weeks following the neurotoxin lesion (53.1% vs 39.4% increase in lesioned vs. control animals body weight, respectively; P < 0.005, Fig. 6a). The parametrial and retroperitoneal fat weights were also increased by 45% (P < 0.02) and 90% (P < 0.001) respectively following peri-SCN dopaminergic neurotoxin administration compared with vehicle infusion (Fig. 6b, c). Daily food consumption over the 16 week study period however was not significantly changed by such lesion treatment (18 g/day vs 17.5 g/day in lesioned vs. control animals, respectively; P = 0.9).

**Fig. 7:**
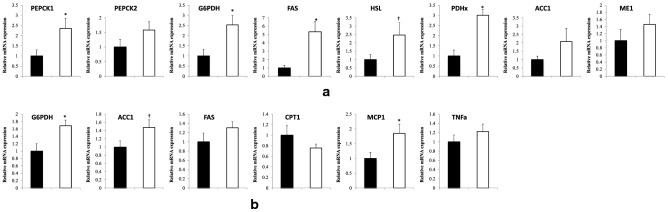
Peri-SCN area dopaminergic lesion increased adipose and liver lipogenic enzyme mRNA levels. Animals received an infusion of 6-OHDA (2–4 μg/side) at the peri-SCN area bilaterally, data were collected 17 weeks post-lesion. **a** Peri-SCN area dopaminergic 6-OHDA lesion (n = 14) impact on mRNA expression for the following genes in the adipose tissue vs vehicle (n = 8): PEPCK1, PEPCK2, G6PDH, FAS, HSL, PDHx, ACC, ME (**b**) Peri-SCN area dopaminergic lesion impact on mRNA expression for the following genes in the liver: G6PDH, ACC, FAS, CPT1, MCP1, TNFa. Data are represented as the mean ± SEM. * P < 0.05 (2-tailed Student’s t-test) or † P  < 0.05 significant difference between treatment groups (1-tailed Student’s t-test)

Analysis of lipogenic enzyme gene expression within the parametrial adipose tissue revealed a marked increase in mRNA expression for several enzymes that facilitate lipogenesis, including pyruvate dehydrogenase complex x (3.0 fold, P < 0.001), glucose 6-phosphate dehydrogenase (2.5 fold, P < 0.02), phosphoenolpyruvate carboxykinase 1 (2.3 fold, P < 0.04), fatty acid synthase (5.3 fold, P < 0.01), and for the enzyme that facilitates the rate limiting step in lipolysis, hormone sensitive lipase (2.5 fold, P < 0.05, 1-tailed) (Fig. 7a).

At 16 weeks following peri-SCN area dopamine neuronal input neurotoxin lesion, hepatic glucose-6-phospate dehydrogenase and Acetyl-CoA carboxylase 1 mRNAs were elevated (68% P < 0.02 and 47%, P < 0.05, 1-tailed, respectively), as was MCP1 mRNA (84%, P < 0.05) versus controls (Fig. 7b).

**Fig. 8:**
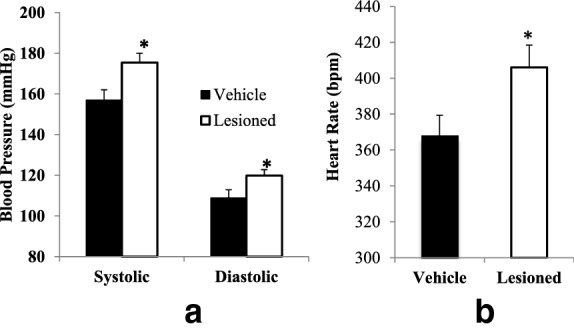
Peri-SCN area dopaminergic lesion induced increases in blood pressure and heart rate. Animals received an infusion of 6-OHDA (2–4 μg/side) at the peri-SCN area bilaterally. The systolic and diastolic blood pressures (**a**), and heart rate (**b**) were measured in rats at 16 weeks after 6-OHDA lesion (n = 14) or sham lesion (n = 8) to the peri-SCN area. Data are represented as the mean ± SEM. *Significant difference between treatment groups (P < 0.05, unpaired Student’s t-test)

Hemodynamic studies revealed that peri-SCN area dopaminergic neuron lesion simultaneously increased systolic blood pressure (from 157 ± 5 mmHg to 175 ± 5 mmHg, P < 0.01, Fig. 8a), diastolic blood pressure (from 109 ± 4 mmHg to 120 ± 3 mmHg, P < 0.05, Fig. 8a), and resting heart rate (from 368 ± 12 to 406 ± 12 beats per minute (BPM), P < 0.05) relative to control animals (Fig. 8b).

## Discussion

The present study is the first to demonstrate in any species that 6-OHDA lesion of dopamine input neurons to the peri-SCN area in young healthy rodents induces simultaneous increases in measures associated with elevated sympathetic tone including elevated resting heart rate, systolic and diastolic blood pressures and circulating norepinephrine levels concurrent with induction of insulin resistance, glucose intolerance, and increased body fat stores without altering food consumption. The peri-SCN and SCN are known to contain both dopamine D1 and D2 receptors and dopamine input neurons to the SCN area and SCN arise from several brain centers, most prominently from the supramammilary nucleus, and a concurrent, coincident circadian rhythm of dopamine release and dopamine receptor availability at the SCN area (each with daily peak expressions at the onset of locomotor activity at light offset) give rise to the circadian rhythm of dopaminergic acitivty therein [24]. Consequnetly, the methodological approach applied herein is appropriate for assessment of functionality of *composite* dopaminergic activity at the peri-SCN area (via abolishing input dopaminergic activity across the 24 h period of the day [including the circadian rhythm and peak of dopaminergic activity) therein) in regulating peripheral fuel metabolism and vascular hemodynamics without actually damaging the SCN itself. Moreover, the selective 6-OHDA lesion approach employed at the peri-SCN area induced chronic, sustained reduction in dopamienrigc input activity therein without destruction of noradrenergic or serotonergic function at the site.

The concurrent increase in resting heart rate, blood pressure, and plasma NE levels strongly suggestive if not indicative of increased *vascular* SNS tone or SNS-parasympathetic balance [11] coupled to the development of the obese, insulin resistant state following this peri-SCN area dopamine lesion of young healthy rats generally observed to be resistant to age-associated insulin resistance [11] and maintained on a low fat diet (18% calories from fat) is intriguing and a major finding of the present study, particularly in light of the substantial breadth of data linking elevated sympathetic tone to insulin resisitance syndrome [13, 30, 31]. Elevated sympathetic tone (including markers thereof such as elevated RHR) both predicts and potentiates future obesity/insulin resistance, type 2 diabetes, and cardiovascular disease in man [13, 30, 31]. Such elevation of SNS tone (and also of RHR) is associated with increased adipose lipolysis, hepatic and muscle insulin resistance, hyperinsulinemia, vascular inflammation, hypertension, obesity, and metabolic syndrome [9–13, 15, 32–35], collectively, cardiometabolic syndrome. The present findings indicate that both elevated SNS tone and many of these cardiometabolic pathologies can be manifested by reducing dopaminergic input signaling to the peri-SCN area. However, the chronological sequence of these events cannot be ascertained from this initial study. Hyperinsulinemia acting within the brain is known to activate a sustained increase of basal SNS tone and chronically increased SNS tone is known to potentiate insulin resistance and hyperinsulinemia [2, 10, 12, 13, 15, 30, 31, 36–40]. While it may be difficult to ascertain the chicken and egg sequence within this positive feedback loop between hyperinsulinemia inducing increased brain activation of SNS tone [37–40] and increased SNS tone inducing hyperinsulinemia and insulin resistance [9–13, 15, 32], the current findings suggest that it may be possible that each pathology shares certain common etiological factors, that include altered SCN clock control of cardiometabolic health as a function of persistent low dopamine input activity to this center. Further support for such a postulate is as follows.

The SCN is a complex bilateral nucleus of neurons within the hypothalamus, whose circadian clock gene expressions are pivotal in the regulation of whole-body physiology in vertebrates [41–44]. Interactions of circadian neuronal activities within the SCN generate target-organ specific SCN output signals via both the neuroendocrine and autonomic nervous systems [45, 46]. SCN output functions are a primary regulator of autonomic balance and several studies have described its regulatory role in modulating autonomic control of visceral metabolism and vascular biology via its hypothalamic(e.g., VMH, PVN) and other brain center interactions [17, 30, 47–49]. SCN ablation and clock gene knockdown studies have identified important roles for the SCN it the regulation of the daily rhythm of blood pressure and heart rate in mammals [20, 23, 50, 51] as well as of hepatic insulin sensitivity, glucose tolerance, and lipid metabolism [21, 22, 52]. However, what input signals to the SCN may regulate its control of vascular biology (and concurrently of metabolism) remain poorly defined and these study results indicate that dopaminergic input signaling to the peri-SCN area plays a role in this regulation [50, 53].

In various studies, the decrease in brain (including SCN) dopamine activity in seasonally, genetically, or dietary-induced obese, insulin resistant animals is coupled to increases in VMH noradrenergic and serotonergic activities as well as to increases in PVN neuropeptide Y (NPY), corticotropin releasing hormone(CRH), and noradrenaline levels [2]. These VMH and PVN neurochemical alterations that associate with diminished brain and SCN dopaminergic input activity in insulin resistance syndrome are known to increase sympathetic drive to the vasculature and heart to increase blood pressure and heart rate, respectively and also to the viscera to increase fat mobilization and hepatic glucose output and lipogenesis [18, 19, 21, 54–56]. Furthermore, when such VMH norepinephrine/serotonin or PVN NPY/CRH/noradrenaline alterations are recapitulated in healthy animals by hypothalamic micro-infusion of these neuromodulators to these sites, these manipulations potentiate hypertension, hyperinsulinemia, hyperleptinemia, obesity, and SNS activation of adipose lipolysis [57, 58] very much as is observed in the present study with SCN dopamine neuron lesion. Moreover, systemic treatment with dopamine agonist to animal models of insulin resistance syndrome reverses the above described aberrant VMH and PVN neurophysiological framework while ameliorating the syndrome [59–61]. The present study findings indicate that the reduction of peri-SCN dopaminergic activity is not merely associated with the cardiometabolic syndrome but in fact can act causaly to facilitate the onset and maintenance of this pathophysiological condition.

Such a neuroendocrine shift driven by such hypothalamic alterations (low SCN dopamine input, elevated VMH NE and serotonin input, elevated PVN NPY and CRN output) can function to facilitate fattening (from hyperinsulinemia) as well as a simultaneous increased adipose lipolysis (from elevated SNS drive to adipose and resistance to the anti-lipolytic effect of hyperinsulinemia) creating a vicious cycle of insulin resistance induced hyperinsulinemia (from elevated FFA mobilization [62]), that supports adipose lipogenesis and central (e.g., hypothalamic) SNS tone activation [9, 11, 63, 64], that in turn drives further adipose FFA mobilization, insulin resistance and hyperinsulinemia. Hyperleptinemia (as observed in peri-SCN area dopamine neuronal lesioned animals in the present study), suggestive of (selective) leptin resistance commonly associated with obesity [65–67], further facilitates increased SNS tone [68] contributing to the insulin resistance syndrome (including hypertension [69]) and compounds the vicious cycle of dysmetabolism [67]. Whether leptin resistance facilitated the obese-insulin resistant condition or was a consequence of this condition or did/was both cannot be concluded from this study. However, previous studies of several experimental animal models of metabolic syndrome suggest a major etiological role for alteration in ventromedial hypothalamic function including increased noradrenergic [57, 59] and decreased leptin function [70] at this center that, importantly, are associated with low brain dopaminergic activity [71, 72]. The VMH leptin resistance and noradrenergic hyperactivity that each induce the metabolic syndrome in animals fed regular chow (as in the present study) occur without any change in food consumption (as in the present study) and result from major changes in energy expenditure/utilization processes [57, 59, 70]. Furthermore, leptin is capable of reducing body weight without altering food consumption [73]. Moreover, systemic treatment of metabolic syndrome animals with dopamine agonist reverses the syndrome and the VMH noradrenergic hyperactivity [71] and appears also to reverse the VMH leptin resistance [72] all without altering food consumption. The present study findings along with these prior investigational results suggest a possible sequence of pathophysiological events in the genesis of metabolic syndrome starting with (stress induced) diminished SCN area dopaminergic neuronal activity that in turn facilitates VMH leptin resistance (and noradrenergic hyperactivity) thus potentiating the subsequent induction of metabolic syndrome with manifest hyperleptinemia in part via a shift in anabolic metabolism towards fat synthesis and accrual (see Figs. 5 and 6) with a possible reduction in total energy expenditure, yet without altering food consumption. Now with the present study findings in hand, further detailed studies of the nature of the involvement of leptin resistance in the expression of the metabolic syndrome induced by SCN-dopaminergic neuronal lesion are warranted. Collectively, these previous and current study observations suggest that loss of the daily dopamine signal to the SCN may act to induce the above described VMH and PVN neurochemical alterations that have been shown to associate with low SCN dopamine input activity and that generate the hypertensive, obese, insulin resistant condition without any requirement of increased caloric intake. While such a vicious cycle as described above appears to have evolved to support survival among animals in the wild against an ensuing long period (season) of low food availability [1, 2], in westernized man such a cycle maintained across seasons of the year over extended time can potentiate cardiometabolic pathology [71].

The peri-SCN area dopamine neuronal lesion induction of the obese, insulin resistant state without alteration in food consumption could be the result of reduced energy expenditure and/or of channeling of anabolic processes towards de novo lipogenesis. Available evidence from seasonal animals indicates that this circadian clock system for the regulation of seasonal metabolism functions in large part to shift anabolic metabolism either towards lipogenesis or protein turnover during the fattening or lean seasons of the year, respectively, without change in caloric intake [1]. Also, seasonal fattening is often unaccompanied by decreased energy expenditure (as in overwintering or migratory vertebrate species) [1]. Likewise in the present study, the observed increased body fat store levels without change in food intake following dopaminergic neuronal lesion at the SCN area over the study period argues for either (1) a shift in metabolic energy utilization towards fat accumulation and away from protein turnover, a phenomenon consistent with (a) the natural seasonal fattening event among vertebrates in the wild and also with (b) an observed reduction in body fat and increased protein turnover without decrease in food consumption with systemic dopamine agonist treatment of seasonally obese hamsters [74], or (2) an actual decrease in energy expenditure coupled to increased lipogenic activity, a phenomenon consistent with observed reduction of body fat and lipogenic enzyme activities and increased energy expenditure with systemic dopamine agonist treatment of leptin deficient obese (ob/ob) mice [60]. We therefore examined the influence of the peri-SCN area dopamine input lesion upon lipogenic pathways in adipose tissue and liver, primary sites for fat synthesis in the rat [75]. Interestingly, such SCN dopamine neuron lesion resulted in a coordinated increase in mRNA levels of several key adipose enzymes each critically involved in fat synthesis.

G6PDH (which provides NADPH for the reduction reactions of fatty acid synthesis), the pyruvate dehydrogenase complex enzyme, PDHx (which provide the substrate acetyl CoA for fatty acid synthesis), FAS (which enzymatically elongates malonyl CoA subunits to produce the fatty acids for triglyceride synthesis), and PEPCK (which provides the glycerol backbone [via glycerologenesis] for triglyceride synthesis [76]) were all markedly increased in adipose tissue from the peri-SCN area dopamine input neuron lesioned vs. control animals. ACC, the rate limiting enzyme for fatty acid synthesis was also markedly elevated by such intervention however its difference did not reach statistical significance. Such a coordinated increase in gene expression among several key enzymatic pathways that cooperate functionally to increase lipogenesis suggests that the dopamine input signal to the peri-SCN area is one that is critically involved in the SCN output regulation of whole-body lipid metabolism and fat store level. Yet, HSL, the key enzyme regulating the release of FFA from adipose was also markedly increased in the SCN dopamine input neuron lesioned group vs. controls. Such findings suggest that the dopamine input to the peri-SCN area regulates (likely via the neuroendocrine axis) adipose metabolism in a manner that maintains (or enhances) responsiveness to the lipogenic effects of hyperinsulinemia (which was also induced by this treatment) yet that allows for resistance to the anti-lipolytic effects of such hyperinsulinemia (manifested in reduced hyperinsulinemia-induced inhibition of HSL expression), likely potentiated by increased sympathetic drive to the adipose [15, 31]. Similar to adipose, mRNA levels of lipogenic enzymes (G6PDH and ACC) in liver were also significantly increased (FAS not significantly). Interestingly, liver MCP1 (a marker of pro-inflammatory macrophages, but also produced by hepatocytes [76]) mRNA was also significantly increased which can contribute to inflammation and insulin resistance [76–79], and hepatic TNF mRNA, an inflammatory cytokine can be produced by MI macrophages and hepatocytes [76] that can potentiate insulin resistance [80–82], was also numerically (but not significantly) elevated. In obesity, adipose insulin resistance respecting insulin’s diminished ability to inhibit lipolysis (in conjunction with increased sympathetic activation of adipose) facilitates an increase in plasma FFA level that in turn is a contributing factor to the genesis of insulin resistance respecting insulin action on glucose balance in liver and muscle [9–13, 15, 32, 62]. In accordance with such a mechanistic pathophysiology, lesion of dopamine input neurons to the SCN area that produced such an adipose biochemical profile also induced the insulin resistant, glucose intolerant state. The present study however does not allow for a time-course analysis of inter-relations between these concurrently observed pathophysiologies. Lastly, it should be noted that neither any possible alteration of the circadian pattern of feeding with greater feeding occurring during the sleep cycle of the day yet without altering total daily food consumption, a perturbation that has repeatedly been demonstrated to induce obesity and hypertension in rodents and humans [83] nor a reduced dopaminergic function-induced reduction in physical activity (as observed with reduced nucleus accumbens dopaminergic activity) [84] were assessed in this study but with the current findings now manifest are worthy of future investigation.

Interestingly, the environmental factors common to the western lifestyle of high fat diet, altered sleep/wake architecture, and social stress are well known to reduce brain (mesolimbic) dopamine activity and alter clock function and also to predispose to insulin resistance syndrome and CVD risk [reviewed in [71]. The present findings suggest that reduced dopaminergic input activity to the SCN can be a contributory etiological factor in the genesis of the cardiometabolic syndrome and that such a perturbation to this endogenous control system for cardiometabolic health does not require a hypercaloric or “westernized” high fat diet for its induction. These data when taken in composite suggest that the diminution of the circadian peak amplitude of the dopamine input signal to the SCN may be at least in part the molecular translation of dietary/stress/altered sleep–wake cycle adverse impact on cardiometabolic health. In agreement with the present findings and the association of diminished circadian peak dopamine activity at the SCN in animal models of insulin resistance syndrome, are clinical studies (1) using Positron Emission Tomography scan stable isotope ligand binding studies identifying a reduction in striatal dopamine binding activity among obese, insulin resistant humans, (2) identifying polymorphs of the dopamine D2 receptor that render it less functional associated with obesity and type 2 diabetes among humans, (3) dopamine D2 antagonist use in humans (though not selective for the dopamine D2 receptor) associating with the obese, glucose intolerant condition [71]. Additionally, pharmacological reduction of brain dopamine activity among young healthy humans by administration of a false dopamine precursor (alpha methylpara-tyrosine) for only a day or two is sufficient to induce insulin resistance [85, 86]. Although these clinical studies could not specifically investigate the target site of the hypothalamus or any subregion thereof in dopaminergic regulation of metabolism, the present study findings suggest that the dopaminergic function at the hypothalamic SCN area may be a particularly important region within the brain for such dopaminergic control of metabolism. Finally, the present study results are consistent with the demonstrated therapeutic utility of circadian-timed administration to type 2 diabetes subjects of a quick release formulation of micronized bromocriptine (a dopamine D2 receptor agonist) (Cycloset^®^), timed to the portion of the day when brain dopamine activity naturally peaks in healthy individuals to improve insulin resistance, glucose intolerance, hyperglycemia and CVD event rate in type 2 diabetes subjects [71].

One final note on the relation of the present study findings to the observations that type 2 diabetes worsens Parkinson’s Disease (PD) disease progression (progressive loss of dopaminergic neurons within the basal ganglia–substantia nigra) [87, 88] is worthy of mention for clarification purposes. Molecular neurobiology studies indicate that each of hyperglycemia, oxidative stress and insulin resistance of type 2 diabetes contributes to dopaminergic neuron dysfunction that can exacerbate PD by blocking neuronal growth and synapse generation and potentiating inflammation, mitochondrial dysfunction, apoptosis, and amyloid aggregation [87]. However, studies of other brain dopaminergic neurons such as the mesolimbic system and hypothalamus indicate that natural stress (high fat diet, sleep/wake architecture alteration, or psychosocial stress) or experimentally induced diminution of mesolimbic [87] or hypothalamic (present study) dopaminergic activity potentiates insulin resistance and its sequalae. Consequently, environmental influences such as certain behaviors and diet that reduce brain dopaminergic activities regulating metabolism can feed forward to potentiate oxidative stress, inflammation, insulin resisitance, and hyperglycemia of type 2 diabetes that in turn feeds back to potentially damage central dopaminergic neurons including those of the basal ganglia-substantia nigra to contribute to PD progression in susceptible patients. The nature of this potential cyclic interaction connecting disrupted dopaminergic–clock potentiation of peripheral dysmetabolism and subsequent central dopaminergic neuronal damage/dysfunction warrants further investigation.

The limitations of the current study include: (a) the lack of more detailed chronological data on metabolic and autonomic endpoints over the 16 week treatment period so an assessment of the time course between insulin resistance, leptin resistance, and increased sympathetic tone could be made, b) lack of assessment of metabolic rate, behavior, and physical activity level during the study, and c) lack of assessment of protein vs lipid anabolic processes before and following such treatment intervention, though now having the benefit of the current study findings such more detailed studies are warranted.

## Conclusions

In conclusion, selective lesion of dopaminergic input neurons to the peri-SCN area of young healthy rats induces a cardiometabolic pathophysiology characterized by increased heart rate, systolic and diastolic blood pressures and increased plasma norepinephrine levels coupled with obesity, hyperleptinemia, hyperinsulinemia, insulin resistance and glucose intolerance. This cardiometabolic syndrome occurred without any increase in caloric intake of a low fat diet and was associated with a marked up-regulation of lipogenic enzymes in liver and adipose. These findings suggest that dopaminergic communication with the SCN that is diminished in insulin resistant states can be causal in its induction and this induction encompasses hyperinsulinemia, hyperleptinemia, and an over-activation of the sympathetic nervous system, a composite perturbation well known to be associated with and to contribute to cardiometabolic disease [9, 11, 23, 29–32, 89]. Circadian-timed pharmacotherapies for insulin resistance syndrome subjects (e.g., prediabetes, type 2 diabetes) that help re-establish the normal circadian-peak activity of dopamine function at the SCN and improve cardiometabolic disease (i.e., bromocriptine-QR) [71] may do so in part by such activity at the peri-SCN area.

## Data Availability

The datasets analysed in the current study are available from the corresponding author on reasonable request.

## Abbreviations

SCN: Suprachiasmatic nuclei
DA: Dopamine
AUC: Area under the curve
L-DOPA: Levo-3,4, dihydroxyphenylalanine
5HTP: 5-Hydroxytryptophan
SNS: Sympathetic nervous system
ANS: Autonomic nervous system
PVN: The paraventricular nucleus
VMH: The ventromedial nucleus
ARC: The arcuate nucleus
GTT: Glucose tolerance test
6-OHDA: 6-Hydroxydopamine
CSF: Cerebral spinal fluid
DOPAC: 3,4-Dihydroxyphenylacetic acid
HVA: Homovanillic acid
NE: Norepinephrine (NE)
MHPG: 3-Methoxy-4-hydroxyphenylglycol
5-HIAA: 5-Hydroxyindoleacetic Acid
HSL: Hormone sensitive lipase
PEPCK1: Phosphoenolpyruvate carboxykinase
PEPCK 2: Phosphoenolpyruvate carboxykinase 2
FAS: Fatty acid synthase
ACC1: Acetyl-CoA carboxylase 1
PDHX: Pyruvate dehydrogenase complex x
G6PDH: Glucose 6-phosphate dehydrogenase
ME1: Malic enzyme
ATPF1: ATP synthase complex F1
CPT1: Carnitine o-palmitoyltransferase 1
TNFα: Tumor necrosis factor α
MCP1: Monocyte chemoattractant protein 1
BP: Blood pressure
RHR: Resting heart rate
NPY: Neuropeptide Y
CRH: Corticotropin releasing hormone
